# The relationship between schizophrenia polygenic scores, blood-based proteins and psychosis diagnosis in the UK Biobank

**DOI:** 10.1038/s41537-025-00725-8

**Published:** 2026-01-14

**Authors:** Kimberley M. Kendall, Sophie E. Legge, Eilidh Fenner, Peter Holmans, James TR Walters

**Affiliations:** Centre for Neuropsychiatric Genetics and Genomics, Cardiff University, Cardiff, UK

**Keywords:** Psychosis, Genetics of the nervous system, Biomarkers, Psychosis

## Abstract

Despite notable progress in psychiatric genomics, there are no validated blood-based biomarkers for psychosis. Previous studies have failed to establish a link between schizophrenia polygenic scores (PGS) and blood protein levels. We aimed to identify associations between schizophrenia PGS and blood-based proteins, and to determine whether levels of 2077 proteins differ in individuals with psychosis. We analysed proteomic and genomic data from 47,969 participants in the UK Biobank. Association analyses in the 47,678 participants without psychosis (mean age 57.1 years, standard deviation 8.1 years; 54% female) identified nominal associations (*p* < 0.05) of schizophrenia PGS with 102 proteins. Four of these (TMPRSS15, ADGRB3, CEACAM21, and KLK1) met the false discovery rate (FDR) threshold of < 0.05. We investigated the association of these four proteins with psychosis in a matched case-control sample (283 cases, 849 controls, mean age 56.9 years, standard deviation 8.4 years; 48% female). In individuals with psychosis, we observed significantly lower levels of KLK1, even after adjusting for potential confounders (effect size −0.25, SE 0.09, FDR 0.049). This direction of effect was opposite to that observed in the primary analysis of individuals without psychosis (effect size 324.67, SE 48.32, FDR 3.85 × 10^−8^). The effect of antipsychotic medication did not explain this difference. This protein should be taken forward for further study and validation to investigate its potential as a psychosis biomarker.

## Introduction

Psychosis is an umbrella term for a group of psychiatric disorders characterised by alterations in perceptions, experiences, thoughts, and behaviour. Psychotic disorders, such as schizophrenia and schizoaffective disorder, are highly heritable^1^, with complex genetic architecture. For example, genetic risk for schizophrenia is conferred by rare variants of large effect, and common variants of individually smaller effect, known as single-nucleotide polymorphisms (SNPs), with associations identified at 287 common variant loci^2^.

Despite considerable advances in psychiatric genomics, there are currently no validated blood-based biomarkers for psychosis. In contrast, biomarker discovery in Alzheimer’s disease has brought considerable benefits, enabling improved diagnosis, prognosis prediction, patient stratification, and therapeutic development^3–5^. Numerous studies have reported alterations in blood-based proteins in psychotic disorders, particularly those involved in immune and inflammatory processes^6–11^. However, it is unclear whether these changes reflect genetic liability, disease pathology, or treatment effects.

The limited research investigating associations between schizophrenia genetic liability, indexed by schizophrenia polygenic scores (PGS), and circulating protein levels has reported either no associations or associations restricted to a narrow set of routinely tested immune and inflammatory markers^6,12^. Previous studies may have failed to establish broader associations due to the small size of available proteomic datasets, thereby limiting statistical power to detect modest effect sizes expected for schizophrenia PGS-protein associations^6^. Analyses in the UK Biobank have also focused mainly on standard haematological and biochemical markers rather than a large-scale panel of proteins^12^, further limiting the ability to detect protein-specific effects of schizophrenia genetic liability.

In this study, we aimed to identify blood-based biomarkers for psychosis by investigating proteins associated with schizophrenia PGS. We then tested whether levels of these proteins differed in individuals with psychosis compared to controls.

## Results

### Association analyses of schizophrenia PGS and blood-based protein levels

Following quality control procedures, ancestry restriction, the exclusion of related individuals, and the exclusion of 291 individuals with diagnoses of psychosis, we retained data for 47,678 individuals across 2077 proteins. 54% of the participants were female (*n* = 25,745), and the mean age of the participants at the time of sample collection (UKBB first assessment centre visit) was 57.1 years (standard deviation 8.1 years; range 40–70 years).

We performed linear regression analyses to test for associations between schizophrenia PGS and protein levels. Of the 2077 proteins analysed, 102 were associated with schizophrenia PGS at nominal levels of significance (*p* < 0.05, Supplementary Table 1) and four met the FDR < 0.05 threshold (Table 1). In sex-stratified analyses, 132 proteins were nominally associated with schizophrenia PGS in females, and two of these met the FDR < 0.05 threshold (LRRC37a2 and KLK1, Supplementary Table 2). In males, 79 proteins were nominally associated with schizophrenia PGS, of which one met the FDR < 0.05 threshold (KLK1, Supplementary Table 3). Sensitivity analyses excluding participants selected for disease-enriched subcohorts yielded similar results; the direction of effect for the four proteins that initially met the FDR < 0.05 threshold did not change, although two of these proteins no longer met the FDR threshold (Supplementary Table 4).

**Table 1:** Association of schizophrenia PGS with blood protein levels in individuals without diagnoses of psychosis.

Protein	Effect Size (SE)	FDR
TMPRSS15	−148.6 (34.17)	9.4 × 10^−3^
Transmembrane Serine Protease 15		
ADGRB3	64.9 (12.6)	2.5 × 10^−4^
Adhesion G Protein-Coupled Receptor B3		
CEACAM21	134.7 (32.2)	1.5 × 10^−2^
CEA Cell Adhesion Molecule 21		
KLK1	324.7 (48.3)	3.85 × 10^−8^
Kallikrein 1		

*SE* standard error, *FDR* false discovery rate.

Results are shown for associations achieving significance at an FDR < 0.05.

### Examining differences in protein levels in individuals with diagnoses of psychosis

**Fig. 1:**
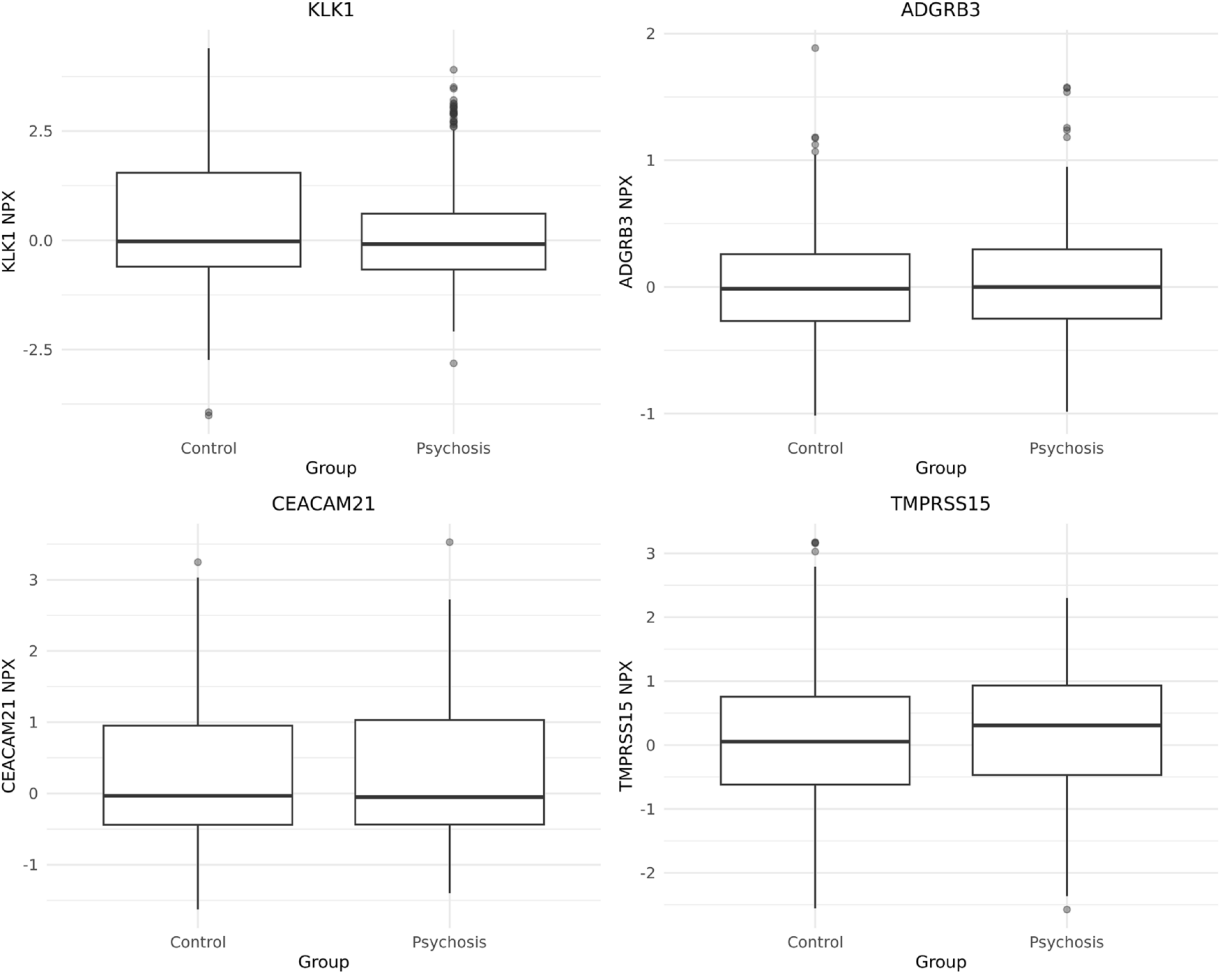
Case-control comparisons of NPX protein levels for TMPRSS15, ADGRB3, CEACAM21 and KLK1 in individuals with psychosis (*n* = 283) and matched controls (*n* = 849). Boxes indicate interquartile range with the median marked; whiskers extend to 1.5 x interquartile range, and points represent outliers. Values shown are raw NPX units - regression analyses were conducted on the matched sample with covariate adjustment as reported in Table 3.

A total of 0.61% (*n* = 291) of our original 47,969 participants had a diagnosis of psychosis. Among these individuals, ICD-10 diagnoses included F20 schizophrenia (*n* = 150), F22 persistent delusional disorder (*n* = 93), F25 schizoaffective disorder (*n* = 38), and F29 unspecified nonorganic psychosis (*n* = 81). These diagnoses were not mutually exclusive, and some individuals had more than one psychosis diagnosis recorded. We examined the association between psychosis diagnosis and levels of the four proteins that met the FDR threshold in the primary analysis to determine if their levels differed in individuals with diagnoses of psychosis compared to controls. We conducted this analysis in a matched psychosis diagnosis case-control cohort (283 cases, 849 controls, Table 2, Fig. 1). The protein KLK1 was present at lower levels in individuals with a diagnosis of psychosis (effect size −0.25, SE 0.09, FDR 0.049). This direction of effect differed from that of the primary analysis, where schizophrenia PGS was associated with a higher level of KLK1 (effect size 324.67, SE 48.32, FDR 3.85 × 10^−8^). We investigated whether this pattern of results may have occurred due to the effect of antipsychotic medication. Medication data were available on 74.0% of the cohort (*n* = 35,508). Of these individuals, 396 were treated with antipsychotic medication (Supplementary Table 5), and 29.3% (*n* = 116) of these had a diagnosis of psychosis. We conducted a linear regression with antipsychotic medication prescription, schizophrenia PGS, and psychosis diagnosis as predictors; age, sex, batch, and time delay as covariates; and protein level as the outcome. For KLK1, both schizophrenia PGS and psychosis diagnosis were associated with protein levels, but antipsychotic prescription was not (Supplementary Table 6).

**Table 2:** A comparison of the demographic information in the cases and matched controls.

	Individuals without psychosis	Individuals with psychosis	*p*
Number	849	283	
Age–mean (SD)	56.9 (8.4)	56.9 (8.2)	0.826
Sex–*n* (% female)	413 (48.6)	136 (48.1)	0.918
Ethnicity–*n* (%)			0.766
White	831 (97.9)	277 (97.9)	
Mixed	6 (0.7)	3 (1.1)	
Other ethnic group	12 (1.4)	3 (1.1)

## Discussion

In this study, we explored the relationship between schizophrenia PGS and blood-based protein levels in the UK Biobank, a large, population-based cohort. Of the 2077 blood-based proteins analysed, we identified 102 whose blood levels were nominally associated with schizophrenia PGS (Supplementary Table 1), four of which met the FDR threshold in analyses adjusted for multiple potential confounders - TMPRSS15, transmembrane serine protease 15; ADGRB3, adhesion G protein-coupled receptor B3; CEACAM21, CEA Cell Adhesion Molecule 21; and KLK1, kallikrein 1 (Table 1). We then examined whether the candidate proteins showed different levels in individuals with diagnoses of psychosis by analysing a psychosis diagnosis case-control sub-cohort from the UK Biobank. One protein, KLK1, exhibited lower levels in individuals with psychosis compared to those without (Table 3).

**Table 3:** Association of blood-based protein levels with psychosis diagnosis in matched case-control cohort.

Protein	Effect Size (SE)	FDR
TMPRSS15	0.11 (0.07)	0.241
Transmembrane Serine Protease 15		
ADGRB3	0.04 (0.03)	0.241
Adhesion G Protein-Coupled Receptor B3		
CEACAM21	-0.01 (0.07)	0.881
CEA Cell Adhesion Molecule 21		
KLK1	-0.26 (0.09)	0.049
Kallikrein 1		

*SE* standard error, *FDR* false discovery rate.

Kallikrein-1 (KLK1) is a member of the kallikrein family of serine proteases. It cleaves low-molecular-weight kininogen to produce bradykinin, a vasoactive peptide which plays roles in vasodilatation, inflammation modulation, neovascularisation, and protection against ischaemic injury in cardiovascular and renal tissue^13^. KLK1 is expressed in a range of tissues, including vasculature, neutrophils, and the central nervous system (cerebral cortex and hippocampus)^14^. To our knowledge, KLK1 has not been implicated previously in schizophrenia by genetic studies, and a review of schizophrenia GWAS summary statistics revealed only a suggestive association with the rs2659058 SNP (Beta 0.01509549, SE 0.0081, p 0.06324)^2^. This protein has not been implicated in schizophrenia or psychosis by large-scale transcriptomic or proteomic studies, although the latter have implicated the kinin-kallikrein system^15,16^. KLK1 has been reported to be neuroprotective in cases of cerebral ischaemia^13^, to decrease depression-like behaviours in rodent models^17^, and to be dysregulated in multiple sclerosis^18^. In addition, the broader family of kallikreins has been implicated in schizophrenia, bipolar disorder, and depression, as well as several neurological disorders, including Parkinson’s disease, Alzheimer’s disease, multiple sclerosis, and epilepsy^19^.

Adhesion G protein-coupled receptor B3 (ADGRB3), also known as brain-specific angiogenesis inhibitor 3 (BAI3), is an adhesion G protein-coupled receptor essential for regulating synaptogenesis and dendritic spine development^20^. Several lines of evidence suggest a role for ADGRB3 in psychiatric phenotypes. Copy number variation studies have implicated structural disruption of ADGRB3 in schizophrenia^21^ with rare biallelic duplications of the gene associated with neurodevelopmental and behavioural disorders^22^; common variant studies have linked this gene to schizophrenia^23,24^, bipolar disorder^25^, lithium responsiveness^25^ and substance use disorders^26^. One study reported altered ADGRB3 expression in the hippocampus of men with schizophrenia^27^, but large-scale transcriptomic and proteomic studies have not suggested a role for ADGRB3 in schizophrenia or psychosis.

Carcinoembryonic antigen (CEA) cell adhesion molecule 21 (CEACAM21) is a poorly characterised protein predicted to act as a cell-surface adhesion and immune-modulatory receptor with potential roles in T-cell activation and immune responses^28^. CEACAM21 was previously implicated in schizophrenia by a family-based GWAS in the Jewish population^29^. However, large-scale transcriptomic or proteomic studies have not suggested a role for CEACAM21 in schizophrenia or psychosis.

Transmembrane serine protease 15 (TMPRSS15), also known as enteropeptidase, belongs to the serine protease family, and its role is to activate trypsinogen to trypsin during digestion^30^. Although primarily expressed in the small intestine, there is evidence of low-level expression in the brain, particularly in neurons under stress conditions. In 2010, Makarova et al demonstrated in a rat model that TMPRSS15 is expressed in hippocampal neurons, where it modulates neuronal survival under glutamate toxicity^31^. Other members of the serine protease family play roles in synaptic plasticity and neuroinflammation^32,33^. SNPs within the *TMPRSS15* gene have previously been reported in GWAS as associated with caudal anterior cingulate cortex volume (psychosis neuroimaging study)^34^, with nucleus accumbens volume in trauma-exposed individuals^35^, and with educational attainment^36^. Examination of the summary statistics from the largest schizophrenia GWAS to date revealed 35 SNPs within the *TMPRSS15* gene associated with schizophrenia at nominal levels of significance (lowest *p* value 1.23 × 10^−3^). For these associations, 28 of the effect directions were negative, and seven were positive^2^. There is evidence for TMPRSS15 playing a role in regulating lithium response in individuals diagnosed with bipolar disorder^37^. However, the protein has not been implicated in schizophrenia or psychosis by large-scale transcriptomic or proteomic studies.

In our study, the direction of the association for KLK1 was opposite when comparing the primary analysis (association of schizophrenia PGS with protein levels) and the follow-up analyses (association of protein levels with psychosis diagnosis). Schizophrenia PGS was associated with higher levels of KLK1, but KLK1 levels were lower in individuals with psychosis. One explanation for this is that the protein may be downregulated in response to the disease process. Another potential explanation is that KLK1 has altered levels in individuals with diagnoses of psychosis due to the effects of medication. We tested this by conducting an association analysis of antipsychotic medication, schizophrenia PGS, and psychosis diagnosis simultaneously with protein levels. Schizophrenia PGS and psychosis diagnosis remained associated with the levels of KLK1, but antipsychotic medication prescription was not associated (Supplementary Table 6). Finally, this result may be due to the interaction of genetic liability and illness status in influencing protein levels. We fitted an exploratory model in the full sample, including a schizophrenia PGS x psychosis diagnosis interaction term and adjusting for the same covariates as in the primary analysis. The interaction term was statistically significant (estimate −1215.0, SE 576.0, *p* 0.0349), suggesting that the association between schizophrenia PGS and KLK1 differs according to psychosis diagnosis status. This result should be interpreted cautiously, given the study’s power and cross-sectional design. Larger longitudinal cohorts will be required to investigate these interactions more robustly.

We used schizophrenia PGS to investigate associations with blood-based proteins in individuals without diagnoses of psychosis. This method is potentially more directly linked to inherited susceptibility than restricting analyses to case-control samples, where outcomes may be influenced by illness status or treatment effects. Earlier studies, including those using similar proteomic platforms (e.g., Olink), did not find strong connections between polygenic scores and protein levels, possibly due to limited statistical power. Our use of a large, well-characterised cohort allowed us to detect small but statistically significant effects. Other studies have demonstrated associations between polygenic scores for complex disorders, including type two diabetes mellitus and cardiometabolic disease, and circulating proteins^38,39^. This suggests that polygenic risk can have broad, system-level proteomic consequences and supports the growing evidence that PGS–proteome analyses can reveal biologically informative pathways. Extending these analyses to psychosis represents an important future direction to determine whether shared or disorder-specific proteomic signatures exist across different forms of genetic liability.

Further research should explore the potential of KLK1 as a biomarker for psychosis. Ideally, this would involve individuals at high risk of psychosis due to genetic factors or those diagnosed with the At Risk Mental State to evaluate the proteins’ diagnostic and predictive value, as well as individuals experiencing their first episode of psychosis with longitudinal follow-up data to determine prognostic utility. A potentially important complementary direction for future research will be to integrate pQTL data with schizophrenia GWAS data, for example, through Mendelian randomisation analyses, to investigate whether circulating proteins have a causal role in schizophrenia risk.

Our results should be interpreted in light of several limitations. *Magnitude of effect sizes and power* - our observed effect sizes were small, and the number of individuals with diagnoses of psychosis in the UK Biobank is relatively low, which may limit power for downstream case-control analyses. Limiting our case-control analysis to individuals with schizophrenia (F20) led to a substantial reduction in the number of available cases (*n* = 145). In this more restrictive analysis, none of our four FDR-significant proteins reached statistical significance, although the effect directions remained the same as in our primary analysis. Larger schizophrenia-specific proteomic cohorts are needed to better understand disorder-specific effects. The schizophrenia PGS explains only a modest proportion of the variance in schizophrenia liability (5–7%)^40^. This limits its potential to explain downstream biological traits, such as protein levels. As a result, true associations are expected to have small effect sizes, and null findings may at least partly reflect limited signal-to-noise rather than a genuine absence of effect. *Generalisability -* individuals with diagnoses of psychosis in this cohort are unusually well functioning–this may limit the generalisability of the results^40^. Our analyses were limited to individuals of European genetic ancestry, so the applicability of these findings to other ancestries remains uncertain. Replication in more diverse cohorts will be crucial to determine the broader relevance of these results. *Replication -* opportunities for replication in independent proteomic cohorts are currently limited. Existing proteomic datasets mainly consist of individuals of European ancestry and often lack linked genomic and phenotypic data at the same scale. Expanding such resources will be vital for future replication and broader applicability. *Temporal relationship of variables* - protein levels were measured cross-sectionally, and in most cases after a psychosis diagnosis was made, limiting opportunities to understand further the relationship between these proteins and psychosis diagnosis (e.g., via mediation analysis). Our findings should ideally be confirmed through longitudinal or experimental studies, with data collected in a manner that allows for the temporal relationship between protein level alterations and diagnosis to be established, thereby permitting causal analyses. *Confounding* – Consistent with previous studies, several demographic, lifestyle, and clinical covariates (e.g., BMI) were associated with protein levels in our fully adjusted models. These expected sources of variation did not affect the interpretation of the schizophrenia PGS protein associations (Supplementary Table 7). Finally, as not all circulating proteins were measured in the UK Biobank release, there may be additional schizophrenia PGS associations that are not included in the current assay panels.

All UK Biobank samples were collected during daytime assessment-centre hours (typically 9 a.m. to 5 p.m.), so between-subject variation is expected to be minimal. However, it cannot be entirely excluded. Additionally, our analytical approach adhered to established UK Biobank proteomic frameworks by applying simultaneous adjustment for pre-specified biological and technical covariates to mitigate a range of potential confounders, including sampling design, technical batch effects, and lifestyle or health-related factors^38,41,42^. Nonetheless, unmeasured confounding remains possible.

Psychotic disorders are heterogeneous, both clinically and biologically, which may dilute or obscure associations between genetic liability and circulating protein levels. Our definition of psychosis captures a broad schizophrenia-spectrum phenotype but does not permit stratification by symptom dimensions, illness stage, duration, or cumulative treatment exposure. Protein levels were measured at a single time point, often many years after diagnosis, meaning that state-related, longitudinal, or treatment effects could not be examined. Such heterogeneity may contribute to the modest effect sizes observed and the lack of consistent direction of effects for KLK1 between the primary and follow-up analyses. Future research should focus on phenotypic and genomic stratification to identify more homogeneous groups with distinct protein signatures. Longitudinal studies with detailed phenotyping will be essential to determine if biomarkers change across illness stages or serve as trait markers linked to genetic risk.

In conclusion, we identified multiple blood-based proteins associated with schizophrenia PGS. We provide preliminary evidence that KLK1 has significantly altered levels in individuals with diagnoses of psychosis, although the direction of effect was opposite to that observed in the primary analysis. These results were not explained by antipsychotic medication prescription. These findings highlight new avenues for understanding the biological pathways connecting genetic liability to psychosis diagnosis and potential biomarkers for future research.

## Methods

### Participants

Individuals aged 40–70 years living in the United Kingdom (UK) were recruited to the UK Biobank between 2006 and 2010. Participants attended UK Biobank assessment centres where they provided detailed health and lifestyle information, underwent cognitive assessments, and supplied biological samples, including blood^43^. The North West Multi-Centre Ethics Committee granted ethical approval to the UK Biobank, and all participants gave written informed consent. This study was conducted under the UK Biobank project number 13310.

### Phenotypic definitions

We defined psychosis diagnosis based on the first occurrence of the diagnosis, as collated by the UK Biobank from death registers, primary care records, hospital admission data, and self-reports (field 130875, accessed March 2025). This included diagnoses of schizophrenia (International Classification of Diseases 10, ICD-10, code F20), persistent delusional disorder (F22), schizoaffective disorder (F25), and unspecified nonorganic psychosis (F29)^44^. This definition captures schizophrenia-spectrum psychoses but excludes affective psychoses.

We extracted a list of antipsychotic medications from the UK Biobank data (field 20003). We used these to create a binary variable indicating whether participants were prescribed antipsychotic medication at the time of their attendance at the UK Biobank assessment centres (Supplementary Table 5). The UK Biobank data field 20003 does not contain dosage or duration data for medication use.

### Genotyping

The UK Biobank participant samples were genotyped at the Affymetrix Research Services Laboratory, Santa Clara, CA. Genotyping was conducted on two arrays, between which there was 95% common content: the UK Biobank Axiom array and the UK BiLEVE array. Standard sample processing was applied and is described elsewhere^45,46^, before imputation using the Haplotype Reference Consortium Panel^47^. Post-imputation QC included a minor allele frequency greater than 0.01, an imputation score greater than 0.8, missingness of less than 0.05, and a Hardy–Weinberg equilibrium *P* value greater than 1 × 10^−6^^48,49^.

### Relatedness and genetic ancestry

We excluded related individuals using the UK Biobank kinship coefficients, removing one individual from each pair with a kinship coefficient ≥ 0.0442 (third-degree relatives or closer). Genetic ancestries were inferred using the UK Biobank array data. The process is described in detail elsewhere^50^ and was based on previously published methods^51,52^. In summary, principal components were computed in the 1000 Genomes Phase 3 reference based on 65,338 ancestry-informative markers. The samples were projected into reference PC space, and the top 34 PCs were input into a Fisher’s Linear Discriminant Analysis model to assign genetic ancestry probabilities. Individuals were assigned a 1KGP-like genetic ancestry label if their ancestry probability equalled or exceeded 0.8^50^. We note that these labels reflect genetic similarity to the 1KGP reference panel and do not represent discrete or biologically distinct categories. All analyses in this paper were conducted on participants with a 1KGP-European-like ancestry probability of 0.8 or higher. The first five principal components from the projection were used as covariates to control for population stratification.

### Polygenic scores

Polygenic scores (PGS) for schizophrenia were calculated based on genome-wide summary statistics from a custom GWAS of schizophrenia from the Psychiatric Genomics Consortium (PGC)^2^ that excluded UK Biobank participants^40^. The PGS were derived using a clumping and thresholding approach, consistent with the PGC, in PRSice-^53^ to ensure comparability with prior schizophrenia PGS work; alternative score-construction methods, such as PRS-CS, were not expected to materially alter the results. Only SNPs with a *p*-value of <0.05 were included; no additional thresholds were tested. Further details are described elsewhere^40^.

### Proteomic measurement, processing, and quality control

The UK Biobank collected blood samples from participants during their attendance at assessment centres between 2006 and 2010. A total of 54,219 UK Biobank participants were selected for proteomic analysis as part of the UK Biobank Pharma Proteomics Project, including (i) 46,595 randomly selected participants who provided blood samples at their initial baseline visit, (ii) 1268 participants from a COVID-19 repeat imaging study, and 6376 participants selected due to specific characteristics, such as case status for coronary heart disease, diabetes mellitus, and chronic kidney disease (groups are not mutually exclusive)^41^. Because the proteomics subsample includes disease-enriched strata, we retained all individuals included in the UKB Pharma Proteomics Project release and adjusted for relevant biological and technical covariates in all analyses, rather than attempting to re-weight or stratify by sampling group. This approach is consistent with prior UK Biobank proteomic studies and ensures compatibility with established analytical pipelines^38,41^.

The UK Biobank processed samples centrally to extract plasma, which was then stored at −80 °C before analysis. Proteomic analyses were conducted on the Olink Explore 3072 platform (Olink Proteomics, Uppsala, Sweden) on standard 96-well plates, each containing up to 88 participant samples, with the remaining wells used for internal controls and bridging samples used for plate-level normalisation and batch quality control^54^. UK Biobank reports the results in normalised protein expression (NPX) units. NPX is an arbitrary unit on a log2 scale–a one-unit difference corresponds to a doubling of protein concentration. These values are relative and internally normalised across samples and plates to allow comparison of protein levels. Samples were processed in batches, with quality control procedures used to mitigate batch effects, including median signal normalisation within each plate^41^. Our analyses used the full combined dataset from the UK Biobank Pharma Proteomics Project Phase 2 release (October 2024), comprising all 53,013 participants analysed on the Olink Explore 3072 platform. Additionally, we excluded (i) participants with more than 60% missing protein values, (ii) proteins with more than 60% missing data, and (iii) proteins with more than 20% of values below the limit of detection. These thresholds were selected to align with common practice in large-scale proteomic analyses and to remove poorly measured proteins or low-information samples while preserving statistical power^38,41^. This resulted in a final set of 2077 proteins for analysis (Supplementary Table 1).

### Statistical analyses

All analyses were conducted on the UK Biobank Research Analysis Platform using R (version 4.4.0). In the primary analysis, we examined associations between schizophrenia PGS and the levels of 2077 proteins. Covariates included sex (field 31), age (field 21003), age squared, time between sample collection (field 53) and analysis (UKBB proteomic files), proteomic analysis batch (UKBB proteomic files), the first five genetic principal components, BMI (field 21001), current smoking status (field 20116), medication (number of medications, field 137), renal function (eGFR calculated using fields 30700, 30720 in addition to age and sex), and liver function (ALT, field 30620). Individuals with diagnoses of psychosis were excluded from these analyses. To account for multiple testing, we adjusted *p*-values using the Benjamini-Hochberg false discovery rate (FDR) correction applied across all 2077 proteins, with a significance threshold of 0.05. We repeated these analyses separately for females and males (Supplementary Tables 2 and 3) and, in a sensitivity analysis, excluded UK Biobank participants selected for disease-enriched subcohorts defined using the UK Biobank data field 30903 (Supplementary Table 4). We repeated the analyses using the same regression models as the primary analysis.

To further investigate the four PGS-associated proteins that met the FDR of <0.05 threshold in the primary analysis, we constructed a matched case-control cohort of individuals with and without diagnoses of psychosis. Matching was conducted using the MatchIt package^55^ in a 3:1 ratio, based on age, sex, ethnicity (field 21000), interval between sample acquisition and analysis, current smoking status, BMI, and number of medications (283 cases, 849 controls, Table 2). The matched controls were selected from within the same UK Biobank sample (i.e., from the pool of individuals without a psychosis diagnosis remaining after all exclusions) and were therefore not an independent cohort. We then conducted linear regression analyses with protein level as the dependent variable and psychosis diagnosis as the predictor. We, once again, accounted for multiple testing using the Benjamini-Hochberg FDR method across the four proteins, with a significance threshold of 0.05.

We then conducted follow-up analyses to determine whether antipsychotic medication prescription was associated with the level of KLK1. To achieve this, we performed linear regression analyses with antipsychotic medication prescription, schizophrenia PGS, and psychosis diagnosis as predictors; age, sex, batch, and time delay as covariates; and protein level as the outcome.

## Supplementary information

Supplementary Table 1

Supplementary Table 2

Supplementary Table 3

Supplementary Table 4

Supplementary Table 5

Supplementary Table 6

Supplementary Table 7

## Data Availability

The data supporting this study are not publicly available but can be requested from the UK Biobank (https://www.ukbiobank.ac.uk/use-our-data/).

## Code availability

The R code used for the analyses in this paper can be found here: https://github.com/KimKendall/Schizophrenia-PGS-Proteomics-UKBB.
